# Independent surrogacy journeys without agency intervention: an ethnographic study of surrogates’ experiences

**DOI:** 10.3389/fgwh.2026.1833823

**Published:** 2026-07-29

**Authors:** Nancy Anne Konvalinka, Ariadna Ayala

**Affiliations:** 1 Department of Social and Cultural Anthropology, Universidad Nacional de Educación a Distancia UNED, Madrid, Spain; 2 Department of Social Anthropology and Social Psychology, School of Political Science and Sociology, Complutense University of Madrid, Madrid, Spain

**Keywords:** autonomy, entrepreneurship, ethnography, feminism, independent surrogacy journey, reproduction, surrogacy

## Abstract

The research presented inquires into how women in the United States who act as surrogates independently, without using agencies, to help others form their families organize, experience, and understand these independent journeys. Based on ethnographic interviews with ten white, middle-class U.S. women, this article analyzes their choice to undertake independent (non-agency directed) surrogacy journeys in the United States, a subject which has received little specific attention in the literature. Data from in-depth interviews regarding the decision to carry out an independent surrogacy, the knowledge necessary to do so, the lived experience of the journey itself, the relationship between the surrogate and the intended parents in the years afterwards, and the perceived pros and cons of independent journeys (including cases in which the women used an agency in later surrogacies) will be presented. Our findings, which document all the steps of the surrogacy journey, show that these women carry out extensive research into all the aspects of the process, making informed decisions throughout. The further election of these women to entrepreneurialize their experience and work in the sector of assisted reproduction and surrogacy will also be considered. We conclude that these women's desire and capacity to control, themselves, the complexity of the medical, legal, economic, relational, and emotional aspects of the surrogacy journey, maintaining their autonomy, lead them to prefer an independent surrogacy journey for themselves, while informing other women of the pros and cons of this type of journey.

## 1 Introduction

Previous ethnographic research with Spanish families formed through surrogacy in the United States spurred each author, separately, to inquire about the American surrogates’ experiences. We were intrigued to find that not only did many surrogates do repeat “journeys” with the same or other families, but that many, after gaining experience in first and perhaps second surrogacies, dispensed with the protective guidance of an agency, working directly with intended families in later processes. This created new research questions such as: Why did the surrogates decide to forgo using an agency? How did they manage the processes on their own? Were they happy with the results of their decision?

This has to do with these women's cumulative experience and their recognition of their own reproductive and organizational capabilities (1, 2): they feel empowered to choose independent journeys. In this article, we shall explore these experiences, as narrated by women who acted independently as surrogates.

Through the analysis of our data provided by these American surrogates, we hope to better understand their capacity to manage the process on their own. We will explore their explanations in terms of gaining experience and agency in managing their reproductive assistance to intended families, in contrast to popular conceptions in prohibitionist countries such as Spain that universally frame surrogates as impoverished victims of rich people and of exploitative clinics (3). Thus, we will examine the actions and activities that these women carry out, in order to analyze the complexity of the labor they undertake, the efficacy and relational quality with which they carry it out, and the importance they give to ethical, satisfactory, memorable, and positive journeys for all involved.

Whereas there is a massive body of research on surrogacy from fields such as anthropology, sociology, psychology, law, medicine, social policy and administration, much of it specifically dealing with surrogates’ experiences, this research mainly covers surrogacy carried out through agencies, so independent journeys figure only anecdotally in much of the literature.

We will approach this under-researched area of independent journeys through chosen aspects emphasized by our interviewees. The first two sub-sections of our results will discuss how these women came to be surrogates and when and why they chose to carry out independent journeys. The third and fourth sub-sections analyze the matching process with intended parents and the negotiation of the contract. The process of pregnancy, birth, and giving the intended parents their baby are the subject of the fifth sub-section and the diversity of post-birth relationships is considered in the sixth sub-section. Finally, the elements that factor into their views on independent journeys and their decisions to follow this path will be examined. Interpretations and conclusions will be presented in the discussion.

## 2 Materials and methods

### 2.1 Theoretical framework

Academic and feminist debates have examined whether surrogacy should be understood primarily as an exploitative practice or, alternatively, as a mechanism that can foster women's autonomy and advance reproductive justice (1, 4–9). Parry (10) highlights the tensions inherent in framing surrogates’ reproductive labor as inherently exceptional or exploitative, noting that in certain national contexts there are persistent difficulties in recognizing the intimate and affective dimensions of this labor. Other scholars (11–16) have emphasized the dilemmas associated with assessing whether surrogacy arrangements genuinely enhance women's agency and autonomy (17, 18). Some authors argue that any potential reforms should prioritize women's capacity to determine their own reproductive trajectories (6, 19–25).

What the ethnographic literature on surrogacy has made crystal clear is the localness of the culture of surrogacy communities and the importance of taking into account the different moral frameworks within which surrogacy practices take place. As Smietana et al. explain, “The concept of moral frameworks helps us understand how actors use dominant normative imaginaries in their specific locally situated reproductive decision-making” (26). Thus, it is fundamental to keep in mind that surrogacy is not the same process and does not establish the same relationships globally; rather, it is defined and shaped by the participants in each specific context, and its definition and shape may differ between agency-managed journeys and independent journeys (27–31).

One strand of scholarship has conceptualized surrogacy as a form of reproductive labor encompassing multiple dimensions. On one hand, it entails significant physical labor, particularly evident in its clinical and biomedical aspects (26). On the other hand, it involves emotional labor (1, 32–35) in the construction of personal relationships of care (36), framing these women's work within a deeply intimate and emotional domain, and involving a high degree of personal commitment (1, 37).

A second line of research examines the integration of individuals with prior experience in the fertility sector—particularly former gestational surrogates—into the industry, through recruitment by agencies or by establishing independent businesses, thereby transforming experiential knowledge into professional expertise (14, 35, 38, 39). In addition to traditionally regulated professions (e.g., medicine, biology, and law), the sector has attracted entrepreneurial actors and fostered the emergence of new niche occupations (14, 36, 40). Consequently, a continuum emerges in which women involved in surrogacy can leverage their reproductive skills and experiential knowledge to pursue professional trajectories in the industry.

The most specific work on independent surrogacies comes from sociologists and anthropologists working outside of the United States, such as Hibino and Shimazono in Thailand (41), Yang in China (42), and Gibson in New Zealand (43). Of these authors, only Gibson (43) deals with women's reasons for opting for independent journeys, explaining that some women who do not meet the clinics’ or the state's criteria for gestational surrogacy decide on independent traditional surrogacy.

Perhaps the research that sheds the most light on independent journeys in the US is Berend's book The Online World of Surrogacy, covering the period from 2002 to 2013. Because the analysis focuses on the Surrogate Mothers Online (SMO) forum, it does not systematically distinguish between agency-concerted journeys and independent journeys. However, Berend provides a wealth of information about the reasons for and characteristics of these independent journeys. As she explains:

Surrogates are increasingly able to find IPs with the help of lawyers or independently. More experienced surrogate are more likely to opt for an “indy journey” because they accumulated more knowledge about the process. Many women who use an agency for their first surrogacy think of it as a learning experience… (44)

Berend emphasizes the online forum’s role, during that time period, in educating new surrogates in the values and ethics held by the online community, providing the knowledge and tools to allow them to avoid depending solely on the agencies for information and enabling them to make smart choices in their surrogacy journeys:

My findings indicate that for surrogates the Internet does reduce inequality and has effectively eliminated the “information monopoly” agencies used to enjoy. Gaining medical and legal knowledge helps to “level the hierarchical relationships” between agency personnel and surrogates and, more recently, between clinics, doctors, and lawyers on the one hand and surrogates on the other (44).

Using these theoretical concepts as a springboard, we will analyze surrogates’ experience of the physical, emotional, and relationship labor in independent journeys, their motivation for choosing this type of journey, and their perception of its inherent benefits and difficulties.

### 2.2 Methodology

Our separate original fieldwork was based in Berkeley, California, but extended to other states in the US, and was carried out between 2018 and 2022. New, unfunded fieldwork was carried out in early 2026 as well, to broaden the data produced.

The data was produced through ethnographic interviews with a total of 24 women contacted through agencies, through a Facebook post by a well-known agency owner and SEEDS (Society for Ethics in Egg Donation and Surrogacy) member, through personal contacts, and through snowball sampling. Our analysis will focus on the ten women who have completed or are in the process of completing, among them, 16 independent surrogacy journeys. The women we contacted personally or through agency recommendations all agreed to participate in our research and the women who responded to the Facebook post or who came from snowball sampling all contacted us freely. The importance of stating this is its effect on the information provided: all the women had positive (although not necessarily perfect) experiences with surrogacy and are outspoken advocates of surrogacy. Most are also advocates of independent surrogacy journeys for women who have the skills and specific personality characteristics that we shall discuss. This interest in presenting their experience in the best light explains the positive, quite “rosy” picture they paint of their independent surrogacy journeys. Clearly, ten women do not make a representative sample in any sense. The lack of national or state registries for surrogates made it difficult to widen our contacts and obtain a more representative sample with a broader base of diverse experiences.

A limitation of the research is its exclusive focus on surrogates. Although other research in which we have participated has focused on intended families, we have no cases of intended families through independent surrogacy and so cannot contrast surrogates’ perceptions of their experiences with those of intended parents. This would be a fertile direction for future research.

Some interviews were carried out in-person, while others were carried out online due to the widely differing geographical locations of the participants. The original interviews (2018–2022) by both researchers focused on the decision to become a surrogate, the reason for choosing an agency or an independent journey, the matching process and contract preparation, and the experience of the surrogacy journey. The five most recent interviews carried out in 2026 focused specifically on these same topics within the women's experience of independent journeys. The duration of the interviews varied between 45 and 90 min.

The earlier interviews were transcribed “manually” (by listening to the recording and transcribing the interview) and the recent interviews were transcribed using either the Teams transcription application or the Google record and transcribe application.

All participants gave their informed consent to be interviewed and to have the interviews recorded. They were assured that pseudonyms would be used in any publications resulting from the research and that no identifying information would be provided.

The resulting narratives were analyzed through grounded theory (45), creating analytic categories directly from the interviews. Some of the recent interviews were carried out jointly by both researchers, who were thus able to cross-check the validity of the analytic categories with reference to previous interviews. Both read them several times to determine the salient concepts regarding independent journeys, taking note of any coincidences and differences in relation to the characteristics of the woman interviewed and her personal trajectory. The relationship between the women's personal experiences, their assessment of the benefits and difficulties of independent journeys as compared to agency-led processes, and their recommendations for successful independent journeys were also studied. In-depth discussion between the authors and comparison with the literature cited in the theoretical framework have served to maintain analytic rigor.

## 3 Results

In this section, we shall analyze these women's experiences in independent or “indy” (a term they have long used themselves) surrogacy processes. Many of these women had also experienced surrogacy journeys through agencies and so they often compare and contrast these different paths. Some of these women direct or work in or with surrogacy agencies and thus offer comparisons between their independent journeys and the services and facilities offered by agencies. Beginning with how they discovered surrogacy and the motivations they state for becoming surrogates, we shall follow the different stages of the journey: 1) the matching process, 2) the creation of the contract, 3) the embryo transfer or artificial insemination, 4) the pregnancy, 5) the birth and giving the intended parents their baby, and 5) the aftermath, with its fulfilment or lack of fulfilment of expectations for a continuing relationship. Finally, we shall consider the benefits and difficulties that these women discuss in their comparisons of agency-led and independent journeys.

Table 1, “Characteristics of surrogates interviewed with independent journeys” provides basic information about the women who collaborated in our research, information that will help the reader contextualize the quotes that appear in the text. All participants were white, English-speaking American citizens. Their employment and educational levels attest to their inclusion in the middle class or middle-upper class; most had university degrees and those who did not had other post-secondary professional qualifications. No difference was observed in their discourse that could be attributed to either the kind of surrogacy (gestational or traditional) or the type of intended parent family (homoparental, heteroparental, or single parent). Most were married and had their own children. The employment status given in the table was their status at the time of the interview. We must note that we have used the rather general description of employment as management position in an agency, employee in an agency, or non-agency employment including services for agencies in order to protect the identity and privacy of the women who have participated in our research; more specific details, together with the other information in Table 1, could lead to the revelation of their identities. Eight of the ten women provided information on the compensation they received for their independent journeys, which varied between $18,000 and $30,000. It must be remembered that some of the journeys took place over fifteen years before the 2018–2020 interviews, corresponding to the lower end of the compensations, while more recent journeys were better compensated. One notable exception is the woman who carried out her journeys in the state of New York, which only allowed reimbursement for expenses, not compensation, at the time of her journeys. A former surrogate who is now a manager in an agency told us that agencies today are providing a compensation of around $60,000. As we shall see, it seems clear that increasing the amount of compensation is not the motivation for independent journeys. If we consider the case of Claire, who received $18,000 for two early independent journeys and was set for a third agency journey at the time of the interview for $60,000, we must discount maximizing economic benefits as a motive for independent processes.

**Table 1:** Characteristics of surrogates interviewed and their surrogacy journeys. Source: Own data.

Pseu-donym	Age (at most recent inter-view)	Education	Family situation	Resi-dence	Total complete surrogacies (A: agency; I: independent; SI: semi-independent	Failed surro- gacies	Current employment at time of interview
Amy	40–44	Non-university equivalent of Master's Degree	Single mom, later with a partner; 2 children	Oregon	4 SI: matched through agency then went independent; USA; heteroparental; singleton I: same couple as 1; twins I: foreign heteroparental; twins I: USA; homoparental; singleton Total: 6 babies, for 3 families	2	Management position in an agency
Abby	25–29	Completed pre-requisites for nursing school and Certified Clinical Medical Assistant license	Married, 3 children	Kansas/ North Carolina	1 I: USA; heteroparental; singleton Total: 1 baby for 1 family In process of next journey	1	Employee of an agency
Claire	35–39		Divorced, 2 children; remarried same-sex couple, 1 child through circle surrogacy	Nevada	4 I: USA; homoparental; singleton I: same couple as 1; singleton I: same couple as 1; singleton A: foreign heteroparental; singleton Total: 4 babies for 2 families	1, last surro-gacy	Employee of an agency
Jennifer	40–44	Degree in management and policy	Married, 2 children	California	1 I: USA; heteroparental (same couple as previous failed attempt with agency; singleton Total: 1 baby for 1 family	1	Management position in an agency
Julia	30–34	Associate's degree (2 years college)	Married, 1 child (“one and done”)	Nevada	2 A: USA; heteroparental; singleton A: foreign; heteroparental; singleton In process of 3rd surrogacy, independent, homoparental Total: 2 babies for 2 families	1	Employee of an agency
Karen	40–44	Degree in Child Development,, training in birth-related services	Married, 3 children	California	2 A: USA; heteroparental; singleton SI: using a caseworker from the agency she works in; same couple as 1; singleton Total: 2 babies for 1 family	0	Management position in an agency
Kenara	45–49	Degree in communication	Married, 3 children	California	2 I: foreign; heteroparental; singleton A: foreign living in USA; heteroparental; singleton Total: 2 babies for 2 families	0	Employee of an agency
Linda	40–44	Bachelor's degree in Science	Married, 3 children	Maryland	3 A: foreign; homoparental; singleton A: same couple as 1; singleton I: USA; homoparental (friends); singleton Total: 3 babies for 2 families	1	Non-agency employment including services for agencies
Paula	45–49	BA in Music and Certification in Technical Documentation	Married, 1 child	New York	2 I: USA; heteroparental (distant cousin); singleton A: USA; homoparental; singleton Total: 2 babies for 2 families	0	Non-agency employment including services for agencies
Rosa	40–44	Business Administration	Married, 2 children	California	4 A: USA; heteroparental; singleton I: USA; heteroparental, singleton I: USA; same couple as 2; singleton A: USA; heteroparental; singleton Total: 4 babies for 3 families	0	Management position in an agency

### 3.1 Finding surrogacy

The paths to surrogacy in the United States are quite varied. In the late 1980s and 1990s, Ragoné found the following:

When I began my research, I found that many surrogates reported having considered becoming a surrogate for an infertile couple even before having learned that programs for this purpose existed. Most surrogates reported having first learned of surrogacy through the media, in particular through popular daytime “talk shows,” […], or through ads placed in the “personals” section of newspapers, most often those placed by surrogate mother programs; some learned of it through friends, and still others from their husbands (46).

Amy (four successful journeys, three of them independent) heard about it from a surrogate in a birth-related training class she was taking. She explains:

It was a very different experience then, than it is now. And when I started surrogacy, when I looked into it, I didn't even know that agencies really existed. I started with a mental health care provider where I was having a conversation about why I was interested in it. Then I actually found an attorney because that seemed like the next logical step to me. And only after I had been in the world for a while did I realize that agencies existed.

Linda, a four-time surrogate, explained that “it was always in my heart to do surrogacy”, as a way of expressing gratitude for her own family of three children. She went directly to an agency for her first journeys. Claire (four successful journeys, three incomplete journeys), said that she was a “helper” kind of person, fertile and with easy pregnancies. She relates coming to surrogacy as follows:

I had the idea of surrogacy from seeing it on television shows, or, you know, so it was kind of like always a little seed planted. […] It was kind of, I would say, over years, nothing, really, you know, a moment, where I said, I’m going to do this. It just kind of developed over time, and then looking into it, and researching it, what little bit was online on the internet back then.

Karen (two completed surrogacies), after a bad experience with an agency, joined SMO and advertised as an independent surrogate. The intended parents who contacted her lived nearby. After an email and a phone conversation, they met that afternoon for a walk, where both she and her husband saw them interacting with their son. According to Karen, this meeting “was helpful for her husband,” who from that moment on supported her desire to be a surrogate.

Abby is a younger, more recent three-time surrogate, whose first surrogacy was in the early 2020s. She began to think about surrogacy after her third child was born and did a lot of research online and on Facebook groups before talking about it with her husband, exemplifying the tendency to independently research the subject instead of going directly to an agency for information.

### 3.2 Decision to carry out an independent journey

We see, likewise, a variety of paths that lead women to independent surrogacy journeys. According to the information from the SMO forum, Berend found that experience in surrogacy often led women to choose independent journeys:

More experienced surrogates are more likely to opt for an “indy journey” because they accumulated more knowledge about the process. Many women who use an agency for their first surrogacy think of it as a learning experience: ‘you learn SO much from letting an agency show you the ropes the first time. It's a great education in surrogacy.’ (44)

This was the case for Linda, who went through an agency in her first two surrogacies for one family:

Yeah, so my first two surrogacies, which were for the same family, but they both went through an agency. So that's how I met them. I didn't know them before, it's very much the typical journey of not knowing them, meeting, connecting, and being supported through an agency.

She was happy with her agency experience:

…I certainly, the agency that I worked with, I adored them. I think they did a wonderful job. It certainly guided me through that. So, it's not kind of, it's not like I came to it from a negative experience with an agency.

Later, she matched independently with a local couple, but the embryo transfers failed, after which she was a surrogate for friends who had been working with an agency but ultimately decided to go independently with Linda.

As mentioned previously, Amy didn't even realize that agencies existed when she began to match for her first journeys. She interviewed with some agencies but did not like the experience:

I interviewed and actually went through screening processes with a couple of agencies and ultimately didn't enjoy the experience and realized that I did not want an intermediary between me and the intended parents. I am a very autonomous person, and I’m a very independent person. And it just didn't make sense to me at that time to have somebody else telling me how to have a relationship with the family I wanted to carry for. Maybe that's because I’d already gone through the process of talking with mental health care providers and talking to attorneys. And so I maybe was a little better educated than some coming into it. Maybe it's just that I’m a control freak and to manage my own life.

Her first two matches (unsuccessful in the end) were independent, through the SMO message board, while a third (a singleton and then twins) began with an agency but quickly became independent:

And that match started through an agency. And we very quickly into the process abandoned the agency. They were not helpful. And when we did the sibling, we did not involve the agency at all. And then I did two surrogacies after that where I also matched independently.

Claire began three surrogacy journeys that ultimately failed with an independently matched couple and then went on to another independent match with another couple for whom she carried three successful pregnancies.:

We actually went independent. We didn't have an agency. I mean, this was 13 years ago. It was kind of a different time. And there weren't, I mean [name of agency] was around. I didn't know about them.

Abby offered two reasons for deciding to go the independent route in her two journeys (the first, with three failed attempts, was incomplete, while the second was successful). First, she alleged her “type-A personality” (implying a need to be in control), something that Amy and Julia also mentioned. Second, she was concerned about finding intended parents who shared her values. Abby told us:

My type-A personality couldn't really handle someone else navigating the journey for me. On the other hand, I am a Christian, and so I do have more pro-life values. And I find that working with an agency, a lot of times intended parents don't have those same values. And so for me, it came down to my personal belief as well as my termination stance, made it a lot easier for me to find an intended parent on my own rather than going through an agency where it might have taken six months to a year to find a match. It took me two weeks going independently. So those were the two major deciding factors for me.

Abby matched through “a Christian Facebook group specifically for matching in surrogacy” for her first, unsuccessful journey; her second match was also a Facebook group, but not specifically Christian-oriented.

Jennifer's case (two surrogacies) was different. Having carried out several unsuccessful pregnancies for a couple with fertility problems, who then ran out of viable embryos, she offered to use her own eggs in a traditional surrogacy arrangement. The agency they had been working with no longer wanted to represent them because she was both the surrogate and the donor. Therefore, they decided to embark on an independent journey together, achieving a successful surrogacy process:

The clinics didn't want to be involved so we did it on our own (old school), with syringe and the father's sperm. We stayed local to each other. We did it and it worked.

This case parallels the situation described by Gibson (43) for New Zealand, where government regulations do not allow traditional surrogacy and so the women who wish to do this type of surrogacy opt for independent processes.

In Karen's case, her first incomplete experience took place through an agency; however, she was dissatisfied with how the process unfolded and did not feel she had sufficient decision-making power regarding either the family for whom she would gestate or certain organizational aspects that directly affected her daily life. This was partly due to the intended parents being based abroad and the agency being located a ten-hour drive from her home. As a result, she decided to pursue an independent arrangement for her first complete journey.

She connected with others through an online chat room for surrogate mothers, where she became a member and accessed the section where surrogates and intended parents posted advertisements. In her words, “I found a very sweet post from someone who wrote: ‘my parents are looking for a surrogate so that I can have a sibling.’” Physical and cultural proximity, together with the opportunity to meet them alongside her husband, greatly facilitated the process. In her case, the absence of agency intermediation proved effective, allowing her greater control over the situation, including the selection of the family for whom she would gestate and the terms under which the process would unfold.

When Rosa (four completed surrogacies) went independent, she didn't seek to earn more money than she had with her first surrogacy. According to her, she adhered to the figures and the contract she used during the first process, which had been handled by an agency:

Ten years ago, independent surrogacy wasn't a big deal. There wasn't much information on Facebook or in groups I knew of. So I used the same contract I had used previously, and…it definitely wasn't like what you hear now, where many women take advantage of the system and ask for more money. But I didn't know anything about it, and I used the same contract and the same figures.

Another factor in the decision to go independent is the economic cost for the intended parents. Amy mentioned savings of $30,000 to $50,000 pertaining to agency fees when journeys were independent. This idea attracted Linda:

I think I also am, you know, conscientious about the cost of service. I know it is, you know, an agency fee is just a part of it, but it's so… it's a big part of it, and it can, you know, make the difference of people being able to, to do that in a way so that people can move forward with surrogacy is also something that, you know, kind of enticed me.

With compensations going from $10,000 to $35,000 for the women involved in the early 2000s, and $60,000 to $65,000 at present, according to our interviewees, the savings in an independent journey can be substantial.

In any case, and in keeping with Berend's (44) analysis of the SMO surrogacy community which included both agency-mediated and independent journeys, there is heavy emphasis on being knowledgeable and being prepared. As Abby told us:

I am very diligent in doing my research. I did probably close to a year and a half to two years of research before I even made my first post in a Facebook group. So I made sure I knew what the ins and outs of surrogacy was, the dangers to independent versus agency, and what I was getting myself into, and what would be required of me going independent versus with an agency.

The women who collaborated with us spoke of online and Facebook group research, consultations with mental health professionals and lawyers, long periods of learning and thinking about the process, as well as family discussions. Husbands sometimes initially rejected the idea but were brought on board by their wives’ explanations of the process and of why doing this was important to them.

### 3.3 Matching

The process of matching intended parents with prospective surrogates has varied over time in agencies, from the end of the 20th century, when the intended parents looked at the profiles of several surrogates and chose one (46), to today, when it is the surrogates who receive the profiles of the intended parents and choose, as women who have gone through agencies have told us. As we have seen in Berend (44), the first decade of the 21st century saw independent matching taking place on the SMO online forum, with intended parents and surrogates contacting one another when they perceived possible compatibility or their stories resonated. Amy describes the message board posts and interactions at that time:

So you could… advertise as a surrogate or an egg donor. […] and you could also, as an intended parent, put up your own advertisements. And they were very much advertisements like you would find in, you know, a newspaper from when we were much younger than we are now. And so you would go in and you would say, like, I’m (Amy) and these are my basic statistics and here's what I’m looking for in a match. And you would kind of present yourself in the classifieds. And then you could respond to other people's classifieds. And then typically you’d start this conversation through e-mail, which would then progress to a telephone call, typically. And, you know, as you worked through the details, you would decide, yes, you know, we would, do or don't want to work with each other. And then everything else would follow.

The SMO forum, described by both Berend (44) and Amy as a close-knit, safe-feeling community, diminished its activity until its formal end in 2020, giving way to Facebook groups. Amy explains: “And when that shift occurred, a lot of us started geographically oriented groups because at that point, surrogacy was still more localized than it is now.” She speaks of the difference in atmosphere in the Facebook groups and of the importance, at present, of checking social media for inconsistencies to avoid scammers.

Matching through an agency involves the agency determining the initial compatibility of intended parents and prospective surrogates based on interviews and questionnaires, as information from both the literature (44, 46) and from the agency management and staff with whom we have spoken shows. Matching independently involves much more work by the prospective surrogates and, we can assume, by the intended parents.

As we have seen in Linda's case, sometimes women have friends or family with fertility problems and offer to carry their children for them. In these cases, people already know one another. Otherwise, the first step can be either reading intended parents’ messages, on the SMO message board, when it was active, and on Facebook pages, at present, or having their own post offering to be a surrogate answered by an intended parent or parents. In line with what surrogates told Ragoné (46) and Berend (44), women look for a message that speaks to them and that reaches their heart.

One important step that both intended parents and surrogates should, but do not always, take is doing background checks on one another, to discover any possible criminal history and to determine financial solvency. Amy admits to having been very lucky in her surrogacy journeys, in the first decade of the 21st century, because at that time it didn't occur to her to do background checks. Abby, a present-day surrogate, emphasized the importance of the background check step as a protection for the surrogate, holding that independent journeys often go wrong when such important steps are omitted through ignorance or through an overly trusting attitude. Another important step mentioned is psychological evaluations on both sides, with the results made mutually available. Thus, we see that trust is not given blindly. Apart from first impressions and good feelings, there is a very real process of checking out objective facts about one another; each party protects itself this way and creates a firm basis upon which trust can develop.

After initial contact, the creation of a relationship proceeds with telephone contacts and online or in-person meetings, depending on the distances involved. Where agencies would normally already have determined compatibility in certain areas, such as amount of compensation, views on termination, vaccines, and willingness to carry twins (although the tendency today is to only implant one embryo), in independent matches, the intended parents and the woman who will act as the surrogate must do this work themselves, to reach agreements on these issues through what they consistently call “hard” conversations. Thus, the emotional work carried out by both parties is considerably greater in independent matches. As Abby put it:

You are trying to build that relationship. But before that relationship can build, there has to be a level of trust. And remember, there's no legal contract binding you to anything at that point. And so I feel that legal is probably like what I would call, you know, the most contractual, the most what I would call “unrelationship” part of the journey because it's all legal logistics and hard conversations and what can go wrong and what can go right. And so I feel like, even though it can be uncomfortable, it is so important that these steps don't get missed in an independent journey because with an agency, they’re the ones doing these things and you’re kind of on the sidelines so you don't have to worry about that, you can just build on the relationship. But in independent journeys, it is completely up to you to make sure that the steps are being taken to protect yourself.

Getting to know one another before deciding to continue the journey together and having the hard conversations about miscarriages, terminations, compensation, future relationships, and everything that will go into the contract, requires a great effort on both sides. As Abby says, there can be uncomfortable moments during these discussions. Rosa describes this as one of the more difficult features of independent journeys:

I think the financial part should not be discussed between surrogate and couple. It can get awkward—so that is hard if it is an independent journey; obviously an agency helps take care of all of that, but independently, my suggestion is to have somebody handle all of that and not discuss it… In our situation, I think it worked because I’d had previous experience with an agency, so I at least knew what it should be like… they had an amazing clinic, the very same clinic I’d already been to… everything was the same: the contract, the doctor, the clinic – they were all the same. The only difference is that suddenly you’re pregnant and you’re having those conversations on your own, so instead of having my ‘case manager’ perhaps having that conversation for me or advocating for me if they might be… I was having to have those conversations myself. I was very lucky. And we ended up as very good friends, so it all worked out! (Rosa, one agency journey, three independent journeys)

Rosa goes on to explain that, in her experience working in an agency, some women prefer to continue with agency support, refusing proposals of independent journeys:

It was the support they had… lots of women mentioned that, thank you, and that's why they chose to go with us [her agency] rather than go it alone. We had a lot of surrogates approach us, and after they’d been through their first journey with us, they were approached to undertake an independent journey, but they turned it down.

However, as Linda says, this joint work serves to create a close relationship right from the start:

I like the independent from the sense that you really have that closer communication with the intended parents earlier on in the process and are kind of working more as a team getting started, versus like two individual players that eventually, kind of, you know, once you’re pregnant and things are moving forward, and you don't have, you know, you’re not going through the heavy medical treatments any more, then you kind of like sync up and have that like open communication and relationship building. I find that like the independent ones, you really start earlier on with that… that back and forth.

### 3.4 Contract

The legal contract seems to be the defining moment of a surrogacy agreement. There is general agreement, among the women who participated in the research and in the literature, as well as in many agencies, that the surrogate and the intended parents must have independent legal counsel, to ensure a proper representation of the best interests of each in negotiating the contract. Linda related her experience this way:

I already had a lawyer that I worked with the first journeys that is an absolutely amazing lawyer that I worked with then for the independent contracts as well. And for me, that's the really important part, is having that independent counsel. And so having that really locked down was, you know, already somebody that I could go to that I know in the field, that I trust, was really, really important.

In one of Amy’s early surrogacies, when she was less savvy, she related a less-than-ideal experience regarding contract negotiation:

What was also a little unusual, and I think this was just the times, was we started medications before the contract was signed. We did all kinds of things before the contract was signed. That never should have happened. And actually, we were still negotiating until the night before the embryo transfer. And it wasn't great.

Abby notes how awareness of the need for independent counsel has evolved:

And then, of course, looking into the attorney side, making sure that I had my own separate counsel is very important. And I feel that, sometimes in independent journeys, no, more so than it was back then even. More attorneys are very forward, like the surrogate needs her own counsel and the intended parents need their own counsel, but it wasn't so outspoken when I pursued my first journey versus it is now.

If the discussion of difficult issues has gone well and everyone is “on the same page”, the legal agreement confirms these joint decisions. Fundamental matters such as the number of embryos to be implanted, reduction if multiple embryos develop, termination decisions, compensation and foreseeable expenses, the funding of escrow (not all the women mentioned this, although it seems to be a given in the recent surrogacies), as well as other issues whose early negotiation can avoid frictions later on, are included in the contract. Linda mentions issues that she felt were important to include:

I knew that there were certain things that were really important to me, and then also certain things from, you know, my past contract that I had already negotiated out of that. So, like you said, they can be super detailed. A lot of that language of like really over-dictating like diet and activity, all of that, I was like, can we just say, if it's approved by my OB, I can do it. And, you know, like follow medical advice and I’m happy to do that, but I didn't want, like, can't take, you know, like dietary restrictions. All of that felt overburdensome, but that's something that I did bring up in conversations before we really… To be able to travel out of state further along in the pregnancy. That's a big thing that's really heavily dictated in the contract. Maryland is a very small state […], so I wanted just, if I wasn't in risk of delivering, I wanted to be able to travel up until 34 weeks versus 26 weeks… […] And then, really, it's not so much dictated in the contract, but open communication was really important to me. […] …wanting that relationship after the child is born, whatever that might look like, but still being open was really important. And then I think you really have to also be comfortable and be open to financial discussions as well. […] So that that was, I just put that out there, they were on board with it, and then it just goes into the contract. So there's no like surprises when the contract comes.

This provides a sharp contrast to the findings that Medina Plana (47) reports for research carried out between 2016 and 2019 with California surrogates working with agencies. Working with the concept of legal awareness, Medina Plana found that these women did not participate in preparing the contract, did not have a clear understanding of the legal language, were presented with the contract to read at the moment of the signing and perfunctorily asked if they had any questions. They assumed that the contract was correct, that they had no control over the content, and that they simply must accept it to go forward with the journey.

Our research indicates that the case is quite different for independent surrogacy journeys, where the women work directly with the intended parents and their own independent lawyers to negotiate the contract. They are proactive in putting forward the terms they require, both in early discussions with the parents and when formulating the contract. According to Abby:

You really have to be able to stand up for yourself and what you want and clearly be able to articulate those things. And if you can't, maybe an independent journey isn't for you. Maybe having an agency as a mediator is the right way to go for you because in an independent journey, there is no one else besides the attorney that's working with you to tell your side of things and what you need and what you want and what's negotiable and what's not. And you have to be able to have those hard conversations.

As mentioned in the matching process, Rosa found the financial discussion with the intended couple awkward. For Karen (two completed surrogacies), there was some discomfort in her first independent journey:

We had lawyers. Everything was laid out but there was no third party to oversee and make sure that the contract… I did not like that. There was no ill intentions. They weren't checking the legal contract. I had to be the one to say: you are supposed to put money in an escrow account. I had to advocate for myself. It was uncomfortable. I had to advocate for myself.

Karen stresses that these direct discussions can be uncomfortable. Our interviewees have told us that requiring the intended parents to put money in an escrow account, for example, is standard practice both in agencies and in independent journeys today. However, having to tell the intended parents to do this is uncomfortable because it seems to send the message that the surrogate does not trust them; this enters into conflict with the idea of a relationship of trust between the parties. These women insist that they have an obligation to protect themselves and their families. They feel that the strength to remain firm and stand up for themselves when the intended parents wish them to give in on something they hold important is a necessary personality trait for independent journeys.

In fact, Abby once broke a match during the contract phase when the intended parents changed the terms they had already agreed upon:

And I will say that I’ve had a broken match. I actually matched with a set of intended parents and we ended up breaking match in legal […] We would agree to something at the very beginning of the journey verbally, but in the contract, they changed what they wanted. And so that was a big no-no for me and kind of a big red flag. Honestly, if you agree to something verbally with me and then to change it later on now that we’re in contracts, expecting me to just go along because I was in contracts, that was not okay with me. So there were a lot of things like that when it came to travel out of state. They actually changed their termination views and what they wanted me to terminate for, which was a no for me. […] But we ended up breaking match because of those things. […] Escrow was another big one. These intended parents didn't want to fully fund escrow. […]…that doesn't offer me protection as a surrogate and I can't in good conscience move forward knowing that if something catastrophic happened, those funds weren't in escrow and what's to stop them from not funding it.

While the women involved in these independent journeys considered the contract binding, as did those in Medina Plana's (47) research, their legal awareness was completely different. Rather than seeing the contract as a difficult-to-understand legal document, prepared by experts, which they had to accept blindly, these women viewed it as a tool to protect themselves and their families, as a document that set out their parameters and requirements for the journey they envisioned. While they might be willing to cede to some of the intended parents’ wishes, they are generally adamant regarding what they feel are the big issues. And if the intended parents do not agree to their terms or try to renege on agreements, this is considered a “red flag” signaling a problematic future journey, and the match can be broken. As Kenara explained, the contract, rather than being an expression of mistrust, was the basis for a trusting relationship:

I felt very much in control of the decision every step of the way. And we have amazing contracts that protect, so I didn't… I knew that I always have that foundation, that I would, never would get tricked in the middle of it because we had a clear understanding. And I think it was the same for the parents. There is a lot of trust there because we knew that we both had agreed to what the contract says.

These women feel that trust is, therefore, based on clarity, forthrightness, and commitment to agreements, both on their part and on the part of the intended parents.

### 3.5 Pregnancy, birth, and giving the intended parents their baby

The early conversations and mutual understandings independent journeys require seem, in many cases, to make for a solid relationship throughout the journey. As Claire commented, comparing her three independent, traditional surrogacies with her agency gestational surrogacy for an Indian family:

So, I would say, I mean, my relationship throughout the pregnancy was pretty similar. I, maybe a little bit more day-to-day conversation with the two men. And that might have been because we didn't have an agency, so we really talked about every little thing, every step, so we were going through contracts, or going through the pre-birth order for the birth certificate, or you know, every little thing, we had to speak on, and I mean, they were both very involved in the pregnancy…

Geographical distance can limit interaction during the pregnancy to phone and online contact, but can also lead to extended physical co-presence. This was the case for Amy's third successful surrogacy in an international match with a Turkish couple. She traveled to Turkey for the transfer, meeting the couple in-person, and then the couple came to the US:

And they actually moved to the US when I was six months pregnant, and stayed until the girls were four months old before they returned home. So we had a nice long arc of time together. They lived, they moved to the same town.

Other than to express the joy of helping the intended parents create their family and of seeing them hold their babies after birth, most of the women in our research did not emphasize the moment of giving birth or of giving the intended parents their baby. This may partly be due to the focus of some of the interviews being the specific characteristics of independent journeys. It may also be the result of their expectations of ongoing contact and a future relationship, a point which both parties had agreed upon in the matching process.

Jennifer (two children and two completed surrogacies), in management at a surrogacy agency, did explain to us why the moment of handing the intended parents their baby is one of the reasons that leads surrogates to want to help other families:

It is such a beautiful thing to do, and I wish more people would understand that. You can't truly understand it until you do it. I get a lot of times from the parents… when they come to when I meet them to help them find a surrogate: how can a woman help me if she doesn't know me? What drives them? What I tell them is quite common as it is from my own point of view: we have already experienced to be a parent, we know that joy that comes when you hold your child in your hand the first time and the thought of, of giving that joy to somebody else and experience it and seeing it at delivery… that it why there's surrogates that do it over and over again! It is because it is so beautiful to witness and it is so giving, knowing that you did it, so you are just very proud.

### 3.6 Post-journey relationships

As time goes on after the children's birth, the relationships between the surrogates and the intended parents develop in different ways, depending on many factors, among them, geographical distance and personal preferences. Of a total of 13 independent surrogacies for 10 families, the women report maintaining contact with 9 of these families. Claire maintains a close relationship with the intended fathers and their children from her first three independent surrogacies:

We talk and visit regularly. I see them probably at least once a year, in person. Otherwise, we exchange texts, and we’re on each others’ social media. We stay in contact, just like any friends, good friends, would do. They [the children] just call me Claire. Yeah, but they all know that they grew inside my belly, they say that and they talk about it. It's fun. When we go to visit them, we stay with them, my youngest daughter gets along with their youngest, which is also a girl, so they are very close… […] I’ve tucked them in to sleep before, when I say, like, it's like being an aunt…

Years later, Amy reported ongoing relationships with the three families for whom she did independent journeys. She maintains a relationship with the first family, explaining that “I think our relationship transcended the surrogacy”, “it became a friendship”. Despite the distance, one of the closest relationships is with the Turkish family for whom she carried twins. In 2018, some seven years after this journey, she said she talked to the intended mother “almost every week”:

I think it's very common with the Turkish culture, like, once you become family, you’re family. And that was not something that I anticipated happening. You know, I went in with the idea that, however the relationship develops, that's fine. My goal is to provide them with children, or a child, and you know, that's, that's enough for me. But instead, we became a family. And they ended up divorcing, actually. And it was a very difficult divorce, and so, um, through this whole process we maintained a relationship with each other. I talk to the girls, I don't know, probably, once a month, once every couple of months, and we practice English, and they know who I am and they know their story. The Turkish culture is very shut down. And they did choose to tell their children the story. And to share that. So they know who I am, and we talk about, a little bit. I mean, frankly, the girls would rather tell me who their friends are at school and what toys they’re playing with, than talk about their creation story…

Similarly, she says she talks to her last intended parents “at least once a week, actually we text back and forth all the time and are pretty involved in each other’s lives”.

Paula values living about eight hours by car from her most recent intended family, a distance she considers relatively close. She talks to them every couple of weeks and does face-time with their almost-one-year-old daughter. She counts on visiting in the future, and told us that an ongoing relationship was an important factor in matching.

Relationships following birth, then, can be more or less intense, from occasional calls or exchange of cards and gifts marking milestone dates, to frequent interaction and involvement in everyday life, with visits where people stay in one another's homes and the families spend time together. This is discussed early on in the self-matching process as a projection toward the future that is fundamental to both parties; this contrasts with journeys through agencies in our broader research, where post-birth contact is much more variable, from none at all to intense ongoing relationships.

### 3.7 Comparing agency versus independent journeys: how to decide

Overwhelmingly, the participants in our research not only had carried out multiple surrogacy journeys, but also worked in what is called the “surrogacy industry” in different capacities, such as facilitators, intended parent accompaniment, case managers and even agency owners and managers. We are not suggesting that this is the case for all surrogates; rather, we find that the women who are the most passionate advocates of surrogacy often wish to continue in the area once their own careers as surrogates are over, and are the most responsive to research calls on the subject. This creates an obvious displacement of the range of their responses on a scale of dissatisfaction to satisfaction with surrogacy in general and with independent journeys in particular.

Whereas a few of these women had only done independent journeys, most had experience with both agency processes and independent processes. Curiously, despite being employed in, or heading, agencies, they did not systematically advocate for using an agency. Some referred to their own “type A personalities” as the driving factor in their own choice of independent journeys. As Amy put it:

So, um, you know, for me, as a type A person, I liked doing it independently. Cause I got to control everything. I didn't ask somebody else what was going on. I always knew. Um, but for the people who aren't that way, having an independent, or, or not independent, but having a third party to help facilitate each of those things, made it a smoother process and sometimes allowed whatever tension there was to dissipate.

Abby also attributed her choice of an independent journey to her “type A” personality:

And I'm very type A. And so for me, there were two, there were two kinds of aspects why I decided to pursue independently rather than through an agency. First of all, I am very diligent in doing my research. I did probably close to a year and a half to two years of research before I even made my first post in a Facebook group. So I made sure I knew what the ins and outs of surrogacy was, the dangers to independent versus agency, and what I was getting myself into, and what would be required of me going independent versus with an agency. […] My type A personality couldn't really handle someone else navigating the journey for me.

This need to independently manage the details of their journeys may also have been a factor in some of these women's careers in agencies. Ayala Rubio (9), in her study of U.S. gestational surrogates, argued that women who choose to become surrogates perceive themselves as autonomous and reflexive individuals with strong research skills. Furthermore, the fifteen women interviewed in her study described themselves as efficient and highly organized, demonstrating a strong capacity to manage multiple activities in their daily lives while successfully reconciling these with their family and social commitments. Interestingly, both the findings by Ayala Rubio (9) and those of the research presented here, contrast with the psychological profile provided by some psychologists who evaluate surrogates for agencies (48, 49), who describe them as women with low self-esteem, who seek a feeling of achievement and being special, who enjoy being pregnant, and may be a bit “unorthodox”. Riddle says that:

In the United States, the profile of a “typical” GC is a woman who loves being pregnant, desires a feeling of accomplishment, and who has compassion for the childless. She may be motivated by financial incentive, but that appears to be secondary to altruism [25] (49).

The divergence between the psychological profile could, on one hand, stem from the varying situations and objectives of a psychologist providing an evaluation for an agency and of a woman explaining her motivations and self-understandings regarding her journeys to an anthropological researcher. As Riddle (49) suggests, women hoping to pass a psychological evaluation to become a surrogate may tend to present themselves in overly positive and self-confident terms; and the same may have occurred in our ethnographic interviews. On the other hand, we must also take into account that most surrogacy is done through agencies, so the bulk of psychological evaluations of surrogates are for agency referrals for prospective surrogates. So the divergence could also be due to the differences between women who choose a journey with an agency and women who choose an independent journey. This is, in fact, what the women who collaborated in our research emphasized: that independent journeys were only for women with great confidence in their ability to manage the process and to maintain firm stances on their beliefs and requirements when necessary. This seems to be what these women describe as a “type A” personality.

When the participants in our research talk about what is important for successful independent journeys, they emphasize two aspects: preparatory research and taking all the necessary steps, with no shortcuts. The requirement of doing the research, or recriminations for not having done it, were a constant on the SMO website, according to Berend (44), and this continues to be the case. We have already seen the many months of research Abby did before her first surrogacy, and she insisted on its importance:

I advise a full year of research for any surrogate looking to go independent. Please do a full year of research. You don't even need to post in any Facebook group, if you join Facebook groups that are educational around surrogacy, you will see every single bad thing that can happen and you’ll see a lot of good stories, too. But I mean, because it's social media, you’re going to see bad things more often than good things. But the bad things teach you. Okay, this happened to someone else. I know in my journey, I want to protect myself against this. What am I going to do? Talk to other people that have been through independent journeys. Okay, how did your journey go? What are things that are non-negotiable for you? What are things that you think need to happen? […] And I don't think anything less than a year gives you enough time to fully understand what you’re going to go through as a surrogate, doing an independent journey and the education that otherwise an agency would provide to you. So do your research. Don't skip steps. That's my best advice.

Experience also plays an important role, when women first become surrogates with agencies and then feel they have the know-how to go independent. Julia, for example, after two surrogacies with an agency and then working in an agency with surrogates, with intended families, in matching and as a case manager, was confident that she could manage her third journey on her own. While Linda feels that the best basis for an independent journey is a previous relationship with the intended parents -in her case, they were friends-, she also said that her experience being a surrogate in an agency and working in informing prospective surrogates has provided her with the necessary skills for an independent journey.

Abby speaks to the second requirement, taking all the necessary steps:

I think where the danger comes from is skipped steps. Don't skip steps. Whether you’re with an agency or independent, the steps are the same. The only difference is you don't have an agency or a third party mediating those and telling you what needs to be done when. So make sure medical records are reviewed. Make sure background checks happen. Make sure both parties are getting psych done. Make sure both parties have independent legal counsel and make sure escrow is fully funded. Don't skip steps just because you’re in an independent journey.

Although we do not have full information on the women's compensation for the different surrogacies they carried out, there are indications that one consideration was reducing the cost for the intended parents by doing the work that an agency would have charged for themselves. As Abby explained, “But when it comes to agencies, between an independent journey and an agency journey, you’re saving the agency fee, essentially, which is usually between 20 and 40,000 on average.” As we have seen, Linda and Amy also discussed the savings por intended parents as one of their own motivations for independent journeys.

Jennifer, who went independent in order to use her own eggs in a traditional surrogacy, presents a slightly different case. She explained that, given the strong relationship she had already developed with the couple and her genuine desire to help them form a family, she never considered increasing her compensation beyond what had previously been agreed upon. Although her husband did suggest that she should charge more for acting as both donor and gestational carrier, she maintains that she never considered increasing her fee.

These comments clarify that increasing their own compensation by charging for the work an agency would normally do was not the common practice for these particular women. The “do-it-yourself” attitude works to save money for the intended parents who are framed not as rich people who can pay exorbitant compensations, but rather as people on a similar socio-economic level as themselves, whom they can help to save money while still receiving a fair compensation. This is consistent with Berend's (33) analysis of independent surrogate's concern about keeping costs down for their intended parents.

Clearly, the women who participated in our research suggest that independent surrogacy journeys are not for everyone. Doing research beforehand, knowing the necessary steps and not skipping any, are major factors. Some women pointed out the relatively recent availability of information about surrogacy on internet as facilitating independent journeys. Other favorable situations for independent journeys are having done previous journeys through agencies, thus learning the ins and outs of the process, or being kin to or friends with the intended parents. As women who have both been surrogates and worked in agencies, they underline the importance of each prospective surrogate choosing the path that is right for her, either with the support of an agency or taking charge of the process on her own, with the legal counsel she has chosen. The path chosen depends on factors such as the time available to dedicate to all the aspects an agency would normally manage, on the knowledge accumulated through research and/or experience in previous journeys with or without an agency, and on the confidence each woman may or may not have in her ability to manage the medical, legal, and emotional components of the process. In the end, the choice depends on the preference for agency support in these issues or self-management.

Two of the women interviewed did independent journeys first and then chose to go through an agency for a later process. Paula explained that her first surrogacy was independent because that was the only path available in New York at that time. By the time of her second journey, surrogacy had been regulated in New York and she was working in an agency, which asked her to be their first surrogate. She agreed and was happy with the experience. As we have seen, Claire's first processes were independent and successful, but she chose to go through an agency for her last journey, describing her reasons for this change as follows:

Um, I definitely felt like, over the years, I’ve seen a lot of other people, I wouldn't say necessarily struggle, but just have a harder time, and I’ve actually heard about some instance where it didn't work out when people didn't go through an agency, and so I knew that this last surrogacy, I wanted to go through an agency, because I wanted that third party to help, kind of support, facilitate, be the middle person to help, you know… the parents. Yeah, and I felt like it worked wonderfully. I would, I’d recommend it to everybody. When I talk to people wanting to become a surrogate, I say, “Please, please, this is your first time, absolutely find an agency and don't try to do this by yourself.”

Thus, circumstances, both external and personal, can influence the choice of an agency or indy journey.

We can conclude that the question is not which surrogacy path, agency or independent, is better, but rather which is the right path for each woman, given her personality and preferences for support or independence, how her personal situation and capacity at a given moment to devote time to all the aspects of the journey, her confidence in her management and research skills, and her experience. The women who spoke with us explained their choice of independent journeys as motivated by a “type A” personality that preferred to control details of the process themselves rather than delegate in an agency and by their confidence in the skills gained through research and experience that enabled them to do this. Saving money for the intended parents was also a concern; independent journeys were framed as eliminating the agency fee, not increasing the surrogate's compensation for doing the extra work. Eliminating the agency fee instead of adding it to their own compensation actually supports the surrogates’ claim that their main motivation is to help people form their families, without giving up what they consider a fair compensation for their reproductive efforts.

## 4 Discussion

It is noteworthy that the participants in our research were almost all surrogates multiple times, with two to four successful surrogacies, as well as other incomplete, failed journeys. We cannot claim that this is statistically significant, there being little quantitative data available on independent surrogacy journeys carried out in the U.S. or in specific states. In addition, our methods of contact, originally through agencies and then through a Facebook announcement, with a subsequent snowball technique of women suggesting that friends contact us, tended to reach women who were content with their experiences as surrogates, women who were then more likely to have repeated this experience. As mentioned in the Methodology section, these characteristics of multiple surrogacies, generally positive experiences, and employment in the sector may influence these women's depictions of surrogacy in a very favorable light. Despite this, several of the women told us that organizing their independent journeys presented a number of challenges, such as having to press prospective parents on contractual matters or discuss financial compensation directly, without intermediaries.

These same women, who often also worked as facilitators, case managers, or owners and managers in agencies, were well aware of everything that could go wrong in any surrogacy. In fact, many of them gathered key information for their independent journeys through their earlier experience through an agency.

We have dealt with a specific subgroup of these women, those who have had one or more independent surrogacy journeys. As they have shared with us, their decisions to forgo agency support stemmed from their confidence that they had the research and/or experience and know-how to protect themselves and their families and negotiate. When they matched independently, they chose the intended parents carefully, placing great importance not only on like-mindedness with regard to the “big issues” such as termination, number of embryos, and compensation, but also on similar interests, activities, and tastes, in an effort to create a trustworthy relationship that would continue into the future.

It is important to emphasize that our results indicate a high level of reflective and investigative capacities among these women, consistent with findings from other studies we have carried out (1, 9, 37). This point warrants further consideration, given the constant media coverage in the Spanish socio-political context that portrays these women as vulnerable and lacking decision-making power, even from some self-proclaimed feminist movements (50). These movements advocate a particular sanctification and protection of women's reproductive capacity, which is invariably linked to rigid representations of femininity, associated with pregnancy and motherhood. These differences serve to highlight the divergent moral frameworks (26) regarding surrogacy in Spain and other anti-surrogacy countries, as compared to the U.S., where these surrogates have told us of their pride in and satisfaction with this process and, in the case of independent journeys, their capacity to direct the journey themselves. Based on our inductive analysis of these women's explanations, we suggest that the importance they place on family in their own lives leads them to want to help others form their own families, while their confidence in their own management skills leads them to take on the organizational work an agency would normally do. By doing this, they blur the boundaries between intimate, family-creating reproductive work and entrepreneurial work.

As we have shown, far from presenting a reified vulnerability, these women demonstrate how they practice self-entrepreneurship, combining their experience, knowledge, skills and legal and medical expertise. They used the expertise of the professionals they chose (doctors, psychologists, and lawyers) to support their journeys. They negotiated the details of their contracts and managed the medical, legal, and economic aspects of their journeys, as well as managing their own emotional work and helping the intended families to have confidence in the agreement and in the women's desire to carry the pregnancy to term and give birth to the intended parents’ baby. Although they acknowledge moments of discomfort in negotiating the contracts, they report minimal conflict during the journey, something they attribute to their good choices in matching, to the sound contractual basis, and to their closeness to the intended parents, as two parties pursuing the same objective: to form a family that could not exist otherwise.

It is essential to highlight the importance these women place on two aspects. First, their ability to initiate the process consciously, with confidence in their own abilities, and with the support of their families. Second, their ability to communicate clearly and respectfully with intended parents, even when it was necessary to set boundaries, determine the financial compensation, or discuss the circumstances under which a decision might be made to terminate the pregnancy. They strongly emphasize that their focus of communication with the intended parents is on establishing a mutual connection that allows them to navigate this journey in a warm and respectful way. This is why they insist that almost all the important conversations were linked to establishing a healthy relational framework, where they could provide the intended parents with the necessary information so that they could be certain there was no doubt that the baby would ultimately be entrusted to them. With the underlying contract as a framework that provides stability to the process, surrogates hope and work to establish a secure relationship, designed to withstand the months of pregnancy and any unforeseen events.

In most cases, the relationship has “transcended the surrogacy”, as one woman expressed it, with a pseudo-kinship/friendship type of relationship continuing many years later. This relationship varies from occasional holiday or birthday cards to monthly or weekly telephone contact, or even reciprocal visits and shared vacation time.

These women shared how they use their expertise, gained through their own research and journeys, to carry out successful surrogacies themselves. But they also put this expertise to use, becoming professionally involved in surrogacy, both during their journeys and once they age out of surrogacy. Case managers, directors of intended parents, and founders of agencies, they become entrepreneurs based on their lived experience in the field. In a somewhat contradictory fashion, they use their expertise gained through both agency and independent journeys to inform, educate, and guide other women in their surrogacy journeys through agencies. They help the prospective surrogates and intended parents that they work with to avoid underestimating the complexity of the process, and they openly acknowledge that independent surrogacy involves risks and uncertainties that are not present in agency processes.

We conclude that these women's desire and capacity to control, themselves, the complexity of the all the aspects of the surrogacy journey, maintaining their autonomy, leads them to prefer an independent surrogacy journey. The satisfaction they express with their journeys, both the agency-led processes and the independent ones, has pushed them to seek work in this sector, work that they will be able to continue when they are past the age for being surrogates. The expertise gained through their own lived experience, especially through their independent surrogacy journeys, provides them with the tools and skills to be successful in this business niche.

## Data Availability

The datasets presented in this article are not readily available because the data come from ethnographic observation and interviews and so contain confidential information which cannot be shared publicly. Requests to access the datasets should be directed to the corresponding author.
